# Racial inequity in grant funding from the US National Institutes of Health

**DOI:** 10.7554/eLife.65697

**Published:** 2021-01-18

**Authors:** Michael A Taffe, Nicholas W Gilpin

**Affiliations:** 1 Department of Psychiatry, University of California, San DiegoLa JollaUnited States; 2 Department of Physiology, School of Medicine, Neuroscience Center of Excellence, and Alcohol & Drug Abuse Center of Excellence, Louisiana State University Health Sciences CenterNew OrleansUnited States

**Keywords:** funding, systemic racism, bias, National Institutes of Health, equity, diversity, inclusion, peer review

## Abstract

Biomedical science and federal funding for scientific research are not immune to the systemic racism that pervades American society. A groundbreaking analysis of NIH grant success revealed in 2011 that grant applications submitted to the National Institutes of Health in the US by African-American or Black Principal Investigators (PIs) are less likely to be funded than applications submitted by white PIs, and efforts to narrow this funding gap have not been successful. A follow-up study in 2019 showed that this has not changed. Here, we review those original reports, as well as the response of the NIH to these issues, which we argue has been inadequate. We also make recommendations on how the NIH can address racial disparities in grant funding and call on scientists to advocate for equity in federal grant funding.

## Introduction

The death of George Floyd at the hands of police officers in the US city of Minneapolis in May 2020 sparked protests across the world and kick-started numerous discussions about systemic racism in the US and elsewhere. During these conversations many industries, professions and workplaces have reflected on the fact that they are built within, by and on a white supremacist culture, which has the effect of assisting white citizens achieve success, while making it more difficult for Black citizens to do the same. These discussions have made it clear that systemic racism permeates nearly every aspect of American society; this includes biomedical research and many of the bodies that fund it, including the National Institutes of Health (NIH).

The NIH funds research at universities and other research institutions in the US through more than 20 Institutes and Centers. In August 2011, a groundbreaking review of grants awarded over a seven-year period revealed that, for independent research grants, applications from white Principal Investigators (PIs) were 1.7 times more likely to be funded than applications from African-American/Black PIs (Ginther et al., 2011). The Director of the NIH, Francis Collins, was quoted at the time as saying: “The situation is not acceptable […] This is not just a problem for the NIH but the whole research community [...] This is not one of these reports that we will look at and put aside” (Corbyn, 2011). However, a follow-up study published in October 2019 showed that little had changed and that applications with white PIs were still 1.7 times more likely to be funded than applications with African-American/Black PIs (Hoppe et al., 2019; see Box 1 for more details about these two studies). Nearly a decade after the first report on the funding gap between African-American/Black PIs and white PIs, it is unacceptable that the NIH has not acted more directly and forcefully to redress the problem.

Box 1. Evidence for racial disparities in NIH funding.

Several analyses have revealed that African-American/Black scientists are less likely to receive NIH grant funding compared with their white, Hispanic/Latinx, or Asian colleagues. In 2011, Donna Ginther of the University of Kansas and colleagues at the NIH and Discovery Logic/Thomson Reuters analyzed new R01 applications submitted for possible funding between Fiscal Year (FY) 2000 and FY2006 (Ginther et al., 2011). The primary finding was that grants submitted with African-American/Black PIs (which were 1.4% of the total sample) were funded at a rate of 17.1%, whereas applications with white PIs (69.9% of total) were funded at a rate of 29.3% (see Table S2 in Ginther et al., 2011). This represented a 1.7-fold advantage for the applications submitted with white PIs over those submitted with African-American/Black PIs. (Technically grant applications are submitted to the NIH by an institution, such as a University, but in practice the PI is usually the major figure creating the proposal.)

   Ginther et al. also used multivariate regression techniques to determine how much of the disparity in grant success could be attributed to PI race/ethnicity as opposed to other factors such as NIH funding rank of the PI’s PhD granting institution, the field of study, prior success of the applicant, the name or perceived prestige of the University the PI works for, the prior study section experience of the applicant, and so on (see Supplementary Materials in Ginther et al., 2011). In short, even when accounting for the effects of factors that might be reasonably expected to (or have been proven to) alter grant success, applications submitted with African-American/Black PIs were still at a significant disadvantage when compared to applications with white PIs.

   A follow-up study by Ginther and colleagues examined the effects of an applicant PI’s publication history on racial funding disparities (Ginther et al., 2018). Differences in the numbers of publications, the number of academic citations of those publications, and the impact factor of the journals in which they were published were used in additional statistical models. Numerous other factors were examined as well, but the bottom line was that only ~25% of the funding gap was explained by supposedly objective measures related to scientific productivity.

   A more recent study by Travis Hoppe and colleagues at the NIH identified and analyzed six stages at which differential outcomes might contribute to an overall difference in funding: how frequently applicants submit; whether an application was chosen for discussion by a study section; impact scores of discussed applications; final funding decisions made by NIH institutes and centers; resubmission rates if the application was not funded; and the topic of the application (Hoppe et al., 2019). They analyzed both new R01 applications and applications to renew R01 grants that were submitted between FY2011 and FY2015. Again, applications with white PIs enjoyed a 1.7 fold advantage in funding success compared with applications with Black PIs, despite the fact that overall NIH success had fallen (from 29.3% to 17.7% for applications with white PIs, and from 17.1% to 10.7% for applications with African-American/Black PIs) in the time between the two studies.

   The title of the paper by Hoppe and colleagues was ‘Topic choice contributes to the lower rate of NIH awards to African-American/black scientists’, even though the abstract states that ‘topic choice alone accounts for over 20% of the funding gap’, which means that ~80% of the gap is not explained by topic choice. Hoppe et al. found that two other factors also contributed to the funding gap – the decision to discuss at a study section, and the impact score – but together they still only accounted for 42% of the gap. In other words, applications from African-American/Black scientists are funded less frequently *even when* research topic and other factors are accounted for, though this does not come across strongly in the title or abstract of the paper, or in the associated press release (NIH, 2019). Interestingly, Hoppe et al. do address the 'Matthew effect' – that is, the idea that prior funding success leads to future funding success, or the rich get richer – at some length but, ultimately, they do not seem to recognize the essential role that it plays.

   There are many more subtle results in these papers that speak to different questions, concerns and proposed explanations. For example, Black PIs are required, on average, to submit a grant application more times before it is funded, and are also less likely to revise and resubmit a previously-reviewed R01 (Table S6 in Ginther et al., 2011) – another double whammy. Furthermore, variables that track with better impact scores for all PIs do not track with better impact scores for Black PIs (Figure S4 in Ginther et al., 2011). And factors related to higher success rates (such as being a Full Professor or publishing with co-authors who publish in the upper quartile of their fields) still fail to close the gap between African-American/Black PIs and white PIs (Ginther et al., 2018).

   An extremely important finding in these papers relates to the fact that NIH does not fund grant applications strictly in the order of the scores awarded by the peer review panels. In the study by Hoppe et al., for example, almost all applications in the top 10% of scores with either African-American/Black or white PIs were funded. However, the lowest ranked funded application from an African-American/Black PI was ranked in the 30^th^–34^th^ percentile range, whereas the lowest ranked funded application from a white PI was in the 55^th^–59^th^ percentile range. Although the number of applications in each five-percentile range was not reported, multiplying the success rate for each percentile bin in Table 1 of Hoppe et al. against the total number of applications per bin suggests that ~119 applications from white PIs with scores in the 35^th^–59^th^ percentile range were funded versus zero applications from Black PIs with scores in the same range (Drugmonkey, 2020a). Remarkably, the number of applications with white PIs funded with scores between 35^th^ percentile and 59^th^ percentile is almost half the number of total funded applications with Black PIs.

## A call to action

This article is a call to action for all of those involved in the biomedical research enterprise, especially for those in leadership positions at the NIH and universities (such as deans and departmental chairs), those involved in the review of grants at the NIH (including NIH staff and researchers involved in the peer review of grant applications), those with tenure or other forms of job protection, those with a seat at the table where decisions are made (for example, members of search and tenure committees), and those who are white or white-passing (and may have access to public or private conversations on these topics that more visually obvious people of color may not). It is time to move the burden of this fight from those most affected by it to those unaffected by it, from those exhausted by it to those that have had the luxury of ignoring it until now, and from junior scientists working to get a foothold in our field to senior scientists that already have one (Dzirasa, 2020a; Odekunle, 2020a).

This action can take many forms, but at the most fundamental level, all of these actions require one to actually… act; not to *think* about acting or *talk* about acting. As Carl Hart, professor of psychology at Columbia University, wrote last year: “Verbal behavior is not actual behavior“ (Hart, 2020). Effective action first requires an admission by individuals and institutions regarding their respective roles in the systemic racism that created this situation – without this simple acknowledgment, it will be very difficult or impossible for scientists to believe the rhetoric about 'doing better'.

Action also requires self-education, and self-education requires time and effort. Some good places to start are curated lists of equity-related articles (Bhalla, 2020), personal academic testimonials (Dzirasa, 2020b; Hart and Cadet, 2020; Odekunle, 2020b) and/or following specific hashtags on social media platforms (such as #blackinSTEM and #blackintheIvory on twitter). Action without self-education may be more harmful than helpful, despite the best of intentions. Self-education on this topic will never end; it requires constant introspection and self-assessment by individuals and institutions, constant commitment to do better, and constant checking of one’s own implicit and explicit biases. In a similar way, action also will never end; it requires constant vigilance for un-level playing fields, threats to racial equity, willingness to call out colleagues and policies that fall short, and work to develop solutions that address these issues.

Nearly a decade after the first report on the funding gap between African-American/Black PIs and white PIs, it is unacceptable that the NIH has not acted more directly and forcefully to redress the problem.

At this point, readers may be asking “What can *I* do to change a system that has existed for 400 years?” We do not claim to have all the answers, but we are confident in stating that systemic change comes from the decisions and actions of individuals - even the longest journey begins with a single step. Find like-minded colleagues, discuss these issues rather than shy away from them, incentivize and value efforts related to these causes, and elevate and provide platforms for the voices speaking out on these topics.

This article has two goals. First, we aim to educate everyone involved in biomedical research about the racial disparities that exist in the funding system and about the negative impact that these disparities have for both African-American/Black biomedical scientists and the field at large. Second, we demand that the NIH address these racial disparities, and we provide possible avenues for doing so.

In pursuit of these goals, we first outline the importance of NIH grants for the careers of biomedical scientists in the US, and review the data illustrating the racial disparity in the federal granting system. We then discuss what the NIH is, and is not, doing in response to this disparity, and finally we suggest several steps they should, and should not, take. It is our hope that these suggestions regarding possible ways forward will educate and empower all stakeholders to demand immediate and effective action from the NIH. Any decisions to address this issue will have to come from the NIH, and to this end it is critical that everyone involved in biomedical research be educated about the racial disparities in grant funding and engage the NIH (and each other) on these issues. Federally funded biomedical research is ultimately paid for by all US taxpayers; it must be ensured that this research addresses the interests and health needs of US citizens of all races and ethnicities.

## Academic career currency

In academic science, research grants are essential career currency, particularly in research-intensive universities – while perceived deficits in teaching and service can often be excused away during tenure review, deficits in research productivity rarely are. In most cases research productivity depends on securing grants because grants fund the research which leads to publications and prestige, and the recruitment of students and postdoctoral fellows. These trainees, in turn, generate more research, more papers and more prestige in a feed-forward loop. Publications are an essential criterion in deciding who is hired into a new Assistant Professor position, who is promoted to Associate Professor, who receives tenure, and who advances to Full Professor. There are also often formal or informal expectations that a university professor supports some fraction of their own salary through grants. It therefore follows that inequities in NIH grant funding rates have real and lasting consequences for the success of academic scientists. If we are to address disparities of opportunity for African-American/Black scientists in academic biomedical sciences research, it is essential to address disparities in funding rates.

It is critical that everyone involved in biomedical research be educated about the racial disparities in grant funding and engage the NIH (and each other) on these issues.

Among biomedical scientists in the US, the most highly sought-after grant is probably the R01 grant from the NIH. This type of grant is considered to be the gold standard by many universities, and other large grants are often referred to as 'R01-equivalent' in promotion criteria. That said, this discussion is not limited to R01 grants, or for that matter to NIH, although much of the data discussed were collected regarding R01 grants from NIH. In all likelihood, these trends extend, to some degree, to other NIH research awards and other funding agencies. For example, the success rate for Black postdocs applying to the NIH for a grant called the K99/R00 Pathway to Independence award is significantly lower than that for their white peers (Pickett, 2018).

One can imagine many intertwined reasons that people experience different rates of success when applying for grants. Among these are the quality of the grant application, prior achievements of the applicant, mentoring received or not received in early career stages, infrastructure to support grant writing, protected time for submitting grants, prestige of institution, the breadth and quality of the network with other scientists, topic choice and specific strategy for seeking funding. Woven into this is the inevitability that early grant success leads to later grant success, or put more simply, that having funding leads to getting more funding. In colloquial terms, the rich get richer. Inevitably, on the other side of that coin, people who do not experience early career success face an uphill battle in achieving success later.

## Trickle down effects of racial inequities in funding

Racial disparities in grant funding reduce the chances that Black scientists will achieve research independence, secure a tenure-track position, be granted tenure, and be promoted to Associate or Full Professor. Racial disparities have many other adverse impacts on the careers of individual Black researchers, but the biggest impact is to reduce the number of Black researchers in the biomedical sciences, which has the effect of reducing racial equity and representation in every university and in every academic field. This leads to a vicious circle which ensures that Black scientists will always be under-represented.

The relative lack of Black scientists may be invisible to a person that is not looking for it, because it is 'the absence of something' rather than 'the presence of something'. This makes it more probable that our institutions will fall for the 'pipeline' fallacy: that is, the mistaken idea that the lack of Black professors is due to a lack of candidates for these jobs, rather than being due to the difficulties that a Black scientist faces when trying to establish a career. This misconception can and does lead to suboptimal solutions that bring new junior scientists into an unchanged system, where they face the same old difficulties. For example, Kenneth Gibbs of the NIH and colleagues have shown that higher numbers of PhD degrees awarded to scientists from under-represented backgrounds does not translate into higher numbers of faculty positions for scientists from these backgrounds (Gibbs et al., 2016).

Having fewer Black scientists in faculty and tenured positions at universities may, and probably does, lead to the following: (i) less attention focused on diversity and equity in recruitment and promotion, as well as other issues that disproportionately affect Black and other minorities; (ii) less promotion of Black scientists (see, for example, Figure S9 in Ginther et al., 2011); (ii) fewer Black scientists in leadership positions (such as journal editors, society presidents, department chairs and university administrators); (iii) less recruitment of Black students into science. Reasons for this include, but are not limited to, less effort to recruit Black students by white faculty, and would-be Black students receiving less exposure to academic science (accelerated by the fact that Black students may be less able to perform unpaid work to gain experience, and by there being fewer role models for Black students to emulate); (v) less sensitivity to issues faced by Black trainees and less commitment to mentoring young Black scientists to independence.

All of this leads to the inevitable conclusion that eliminating the disparity of NIH grant award rates for Black scientists is a critical and pressing issue that, if addressed, will have a large and long-term positive impact on providing equitable access to academic science careers, regardless of race. Efforts by the NIH to respond to the racial disparities revealed by Ginther et al., 2011 and Hoppe et al. in 2019 fall into three categories: first, attempts to explain away the disparities by attributing the funding gap to factors other than the race/ethnicity of the PI; second, attempts to fix the 'pipeline' by funding more African-American/Black trainees; third, attempts to identify and eliminate bias at the level of peer reviewers, with an emphasis on the subconscious, or implicit, biases. However, as we shall see, these responses have been inadequate.

Racial disparities in grant funding reduce the chances that Black scientists will achieve research independence, secure a tenure-track position, be granted tenure, and be promoted to Associate or Full Professor.

## Our response to the response from the NIH

The peer review of most grant applications submitted to the NIH is overseen by its Center for Scientific Review in a two-stage process. The first stage involves review by a Scientific Review Group (usually a study section or special emphasis panel) composed primarily of scientists with expertise in the relevant area of research. R01 grant applications are generally scored on five review criteria: significance; investigator; innovation; approach; and environment (with 1 [exceptional] being the highest score and 9 [poor] being the lowest). The second stage involves review by the advisory council or board of the relevant Institute or Center: these bodies are "composed of both scientific and public representatives chosen for their expertise, interest, or activity in matters related to health and disease" (https://grants.nih.gov/grants/peer-review.htm). Applications that are recommended for approval at both stages have a higher likelihood of being funded, but the final decisions on which applications are funded are made by the directors of the relevant Institutes or Centers. It should be noted that Institutes and Centers differ widely in their funding strategies, such that some fund grants primarily in the order that they are ranked by peer review, whereas others fund grants with lower scores for a variety of reasons that includes programmatic priorities of that Institute or Center.

Much of the response from NIH has come from the Center for Scientific Review (CSR). In June 2020, the CSR Director, Noni Byrnes, wrote a blog post with the title 'Race and Peer Review' (Byrnes, 2020) that we will quote from at length, before responding to the points made by Byrnes:

"As indicated by several published studies over the last decade, and NIH’s own analyses, there remains a serious and disturbing disparity in NIH R01 award rates between white and Black applicants. Isolating the effect of race in the peer review process is a difficult undertaking, since there are many secondary, linked variables (e.g. institutional “prestige”, investigator “pedigree” – who trained with whom, networks, Matthew and “halo” effect, etc.) that are themselves linked to racial disparities in opportunity and access. However, there is absolutely no question that implicit bias exists in all of us as individuals, and the CSR peer review process, with 18,000 unique individuals serving as reviewers, is not immune from these biases.

Since 2019, CSR has initiated a number of efforts to mitigate bias, both at the individual and systemic levels. These are listed below, and as you can see, they are in various stages of development.

1. Development of bias-awareness training modules, with case studies, for reviewers and staff. This is being piloted [...]. Based on feedback from the pilot, we plan to refine and roll out the training to all CSR reviewers and staff in 2021.
2. *The CSR Advisory Council working group to simplify review criteria made a major* *recommendation* *to decouple the science/idea aspects of the review (significance, innovation, approach) from the person-based aspects (investigator, environment). This sets the stage for a de-identified review process for evaluation of scientific merit.*
3. *Along the same lines, CSR is initiating a multi-stage, partially* *double-blinded review process* *for the Common Fund transformative R01 reviews in fall 2020.*
4. We continue our ongoing efforts to broaden the pool of reviewers with respect to career-stage, including doubling the number of early-career reviewers serving on our committees, and actively encouraging recruitment of associate and assistant professors. We know that these cohorts are more diverse in both gender and race/ethnicity.
5. While a vast majority of our 18,000 peer reviewers conduct themselves in a highly ethical manner, we continue our critical efforts to identify and take action against those who manipulate the peer review process. Those involved in or unfairly benefiting from the tampering are rarely women and are almost never from under-represented minority groups.

While these may be some steps in the right direction, we recognize that there is much more that must be done. [We] are planning to hold a series of forums to have a conversation with you about the data on racial disparities in NIH funding, CSR’s plans, and to hear your thoughts. The first forum will be held on July 8, 2020.

Below we give our thoughts and responses to these five points.

### 1: Bias-awareness training

This training may help and has been in process for some time, but frankly, it will not fix the problems being discussed here. There are already academic works appearing that show that anti-bias training does not result in changes to biased behavior (Chang et al., 2019; Glasman and Albarracín, 2006; Kaste, 2020) and more general reviews emphasize a profound lack of empirical support for success (Paluck and Green, 2009). The NIH should not rely (solely) on this strategy to create substantive change.

### 2: Decouple the science/idea aspects of peer review from the person-based aspects

This strategy is unlikely to be workable in practice (see below) but, more importantly, there is reason to believe it would not fix the grant award disparity even if it were possible to accomplish. Data from the NIH show that scores from the initial peer review process are most strongly correlated with Approach and Significance, somewhat less so with Innovation, and are correlated most weakly with Investigator and Environment (Berg, 2010; Rockey, 2011). Therefore, the grant review criterion most closely aligned with the person-based aspects of the application is a weak contributor to the final ranking, and therefore likelihood of funding, of grants.

### 3: Anonymizing applications

Anonymization is also unlikely to fix the issues being discussed here. In a recent study conducted by CSR (and described in CSR, 2020a), 1200 applications were anonymized according to PI name, but redaction was only partially successful (i.e., some reviewers figured out which applications came from which PIs), and scores for applications from African-American/Black scientists did not improve. This undetected breakdown in the anonymization is likely to persist if this strategy is implemented for all applications because PIs work on incredibly niche areas that are easily recognizable according to preliminary data and study design, and because PIs frequently cite their own work in grant applications.

The difficulty of anonymization was further evident in a study in which researchers at the University of Wisconsin-Madison and the University of Arkansas attempted to detect implicit bias by changing the names of PIs in grant applications and having them reviewed by different reviewers in parallel (Forscher et al., 2019). The PIs on the applications were altered with 'white names', such as Greg Murphy and Anne Kelly, and with 'Black names', such as Darnell Washington and Latonya Jackson. The authors of this study, which was funded by the NIH in the wake of Ginther et al., 2011, reported that they "find little to no race or gender bias in initial R01 evaluations". However, they did not systematically determine if reviewers divined the true purpose of the study, and thus did not allow for the possibility that detection of the experimental manipulation of PI race might have influenced how the reviewers scored the applications. Indeed, the study did indicate that some reviewers caught on to their purpose, which makes it difficult or impossible to quantify the role of implicit bias, which was the target being studied.

### 4: Broadening of the pool of reviewers with respect to career-stage

This proposal has some hope of addressing the racial disparities observed in the grant funding process. According to one study, 2.4% of study section members in the period FY 2011–2015 were African-American/Black (compared with 77.8% who were white; figures based on reviewers with reported demographic data; Hoppe et al., 2019). However, in the 2010 census, 14% of US citizens identified themselves as African-American, or mixed race including African-American.

According to the CSR “there must be diversity with respect to the geographic distribution, gender, race, and ethnicity of the membership” of study sections (CSR, 2020b). As far as we are aware, the NIH has not officially stated what fraction of study section members would need to belong to various races and ethnicities for study sections to be diverse. However, CSR director Noni Byrnes has publicly stated that the number of African-American/Black reviewers on a study section should reflect the proportion of African-American/Black scientists in the field, in response to questions during online forums conducted in July of 2020 which were summarized by CSR, 2020a. We have two main problems with this, as outlined below.

First, the underrepresentation of Black reviewers on study sections contributes to lower funding rates for grant applications with Black PIs, and because reviewers tend to be drawn from the ranks of those with NIH grants, these lower funding rates essentially ensure that Black researchers remain underrepresented on study sections. The cyclical nature of the disparities needs to be acknowledged and addressed with direct intervention.

Second, the NIH is a taxpayer-funded body tasked with improving the health of all Americans, and it is our view that targets for diversity should reflect the diversity of the nation rather than the scientific community. In other words, the target should be for 14% of study section members to identify as African-American, or mixed race including African-American.

It is clear that a lower target is a recipe for continuing the disparities of the status quo. If Black scientists cannot succeed within their fields, the numbers of Black scientists will never increase, and therefore the representation of Black scientists on review panels will never increase. With typical panels of ~20–30 reviewers voting on applications and only three reviewers doing in-depth analysis of each application, it is unlikely that low single digit representation of Black reviewers can provide effective balance. The best approach to break this cycle is direct intervention with a target percentage of African-American/Black reviewers that reflects the diversity of the US population. It is important that efforts are made not only to diversify study section standing member rosters, but also *ad hoc* reviewer representation in study section meetings, as well as special emphasis panel meeting rosters.

It is encouraging that the CSR wants to broaden its pool of reviewers, but it is perplexing that it proposes to achieve this only by recruiting younger reviewers. They say they will use this strategy because “these cohorts are more diverse in both gender and race/ethnicity”. However, it is not clear why the policy does not simply instruct the Scientific Review Officers who oversee the peer-review process to recruit more African-American/Black reviewers. It is unlikely that all the mid- and late-career African-American/Black PIs with grants are already reviewing applications for the NIH. One strategy that could be employed is to ask African-American/Black researchers who have submitted proposals that scored in the top half of applications, but were not funded, to review applications. However, no matter what form such an initiative or program would take, it would undoubtedly have positive effects on the funding rates for African-American/Black applicants. It would also give a greater number of African-American/Black scientists much-needed insight into the peer review process, thereby increasing the likelihood that their own applications will be competitive for funding.

### 5: Identify and take action against those who manipulate the review process

This point sounds admirable, and has been of concern across the NIH recently (Lauer and Amero, 2019), but one wonders why this hasn’t always been the case. Moreover, it is not clear how it is related to the review of grant applications with African-American/Black PIs: if the CSR thinks that the peer review process has been manipulated to advantage or disadvantage racial groups, it should be transparent about these concerns.

The NIH must acknowledge that systemic and structural racism exists within its Institutes and Centers, and it must create a plan with actionable items that will have a real and lasting impact on the racial disparities discussed in this article.

## Recommendations to address racial disparities in grant funding

### 1: The need for increased data transparency

The work of Ginther et al. and Hoppe et al. show that it is long past time for the NIH to publish relevant review statistics that pertain to race and ethnicity on a regular basis. The NIH already publishes success rates based on grant mechanism, type code (new grants versus competing continuations), the gender of the PI and the stage of investigator (see https://reporter.nih.gov/). The NIH has more recently issued statistics on per-*investigator* success over, say, a five-year interval which is a welcome addition. Importantly, the data for success rates for R01 grants across the NIH which are reported in the papers by Ginther et al. and Hoppe et al. should be reported for each Institute and Center, and also for other grant mechanisms. Rationales that too few applications are available and would somehow violate 'privacy' of applicants should not be tolerated as legitimate excuses. If this is perceived to be a problem, the simple fix would be to obtain consent from those applicants to include their de-identified applications in the dataset. We are confident that African-American/Black PIs would be happy to do this, but even if they are not, that should be their decision to make.

The CSR should also publish race and ethnicity data for each standing study section on at least an annual basis. This should be accompanied by statistics on reviewer gender, geographic location, career stage and funding status. In order to ensure full transparency and the ability to analyze data over time, longitudinal data reaching back to the creation of specific study sections should be recovered, published and continued to be published with each update. The CSR should also publish success rates for applications and applicants to each study section disaggregated by race and ethnicity. Finally, the CSR should analyze and report the racial composition of special emphasis panels.

### 2: Using paylines to reduce disparities

As mentioned above, it is the director of the relevant NIH Institute or Center who makes the final decision about which grants are funded: the study sections and advisory councils or boards are only advisory. Some Institutes and Centers fund grants primarily in the order that they are ranked by peer review, whereas others do not. In this latter scenario, although applications with, say, a 25^th^ percentile rank are more likely to be funded than applications with a 30^th^ percentile rank, the Institute or Center might decide to fund one or more applications with a rank of 30^th^ percentile and not fund one or more with a rank of 25^th^ percentile (Kienholz and Berg, 2013). Such 'exception pay' decisions can have a significant impact on which applications are funded and which are not. As mentioned in Box 1, for the sample of applications studied by Hoppe et al., one of the present authors (MAT) estimated that approximately 119 applications with white PIs in the 35^th^–59^th^ percentile range were funded, whereas zero applications from African-American/Black PIs with scores in this range were funded (Drugmonkey, 2020a).

It is important to note that only ~1.5% of total applications are submitted by Black PIs: the fact that this percentage is so small is a major problem. However, it also makes the disparity relatively easy to address by reversing or equalizing disparities in discretionary funding decisions. If unsuccessful applications with Black PIs ranked in the ~15^th^–35^th^ percentile range were funded instead of applications with white PIs that were funded despite being ranked outside the top 35% of applications, racial disparities would be reduced and the average standard of funded applications (as assessed by peer reviewers) would improve! This approach would not require formal programmatic change – if discretionary decisions can be made in a way that favors white PIs over Black PIs by a 119–0 margin, then they can just as easily be made in a more equitable way, for example by prioritizing research topics more often proposed by Black PIs. If this is done, the success rate for African-American/Black PIs will increase significantly, while the success rate for white PIs will be reduced by an imperceptible amount (Drugmonkey, 2020a; Drugmonkey, 2020b).

Based on statistical approaches that parse percentile scores into small ranges, then analyze funding rates for white and Black PIs, Hoppe et al. conclude that "final funding decisions by ICs, whether based on impact scores or discretionary funding decisions, do not contribute to the funding gap” (Hoppe et al., 2019). This conclusion is at clear odds with the reality that ~ 119 grants with white PIs were funded with percentile ranks worse than any application funded with a Black PI.

### 3: Using a top-down approach previously used to help early-stage investigators

The NIH has a long history of concern about the low success rates of grant applications from early-career researchers, and initiatives to fix a perceived bias against such researchers date back to at least 1977 (NIH, 2020). In 2007, for example, policies were put in place to enhance the funding of applications with Early Stage Investigator (ESI) PIs, including exhortation of reviewers to be lenient and a relaxed payline that applied only to these applications. It is critical to note that the NIH did not engage in any investigations into the 'real source' of the ESI funding disparity, that it did not propose anti-bias training of reviewers, and that it did not propose that improvements to the training pipeline would somehow solve the problem many years in the future. The NIH simply declared the funding disparity an inappropriate bias in the review process and put in place a set of top-down quota-based affirmative action procedures to redress the funding disparity.

The justification and implementation of ESI initiatives and programs suggest that there is no credible reason why similar types of programs could not be implemented with respect to African-American/Black PIs and other subgroups of underrepresented and disenfranchised PIs. Furthermore, there is no reason why this could not be done immediately.

## Conclusion

It is unacceptable that the racial disparities in the NIH grant funding system reported by Ginther et al. in 2011 were still present during the period studied by Hoppe et al., 2019. It is unacceptable, almost a decade after the original report, that we must await the occasional publication of findings from targeted studies to see if funding rates for grant applications with African-American/Black PIs, and other PIs of color, continue to suffer from a bias. It is unacceptable that programs have been implemented across the NIH to address some disparities (e.g., career stage), but have not been implemented to address racial disparities – this suggests to us that it would be possible for the NIH to address racial disparities in grant funding, and that the failure to do so is a choice by NIH leadership. It is unacceptable that data on racial disparities in funding are often presented in a way that appears to excuse the NIH and blame African-American/Black PIs (e.g., for their choice of research topic). Finally, it is unacceptable that health conditions and topics of interest to Black citizens are systematically overlooked for research funding. It is our duty as biomedical research scientists to demand better. The NIH must acknowledge that systemic and structural racism exists within its Institutes and Centers, and it must create a plan with actionable items that will have a real and lasting impact on the racial disparities discussed in this article.
